# Supplementary material for: The Inhibition of TREK-1 K+ Channels via Multiple Compounds Contained in the Six Kamikihito Components, Potentially Stimulating Oxytocin Neuron Pathways
Source: Int J Mol Sci. 2024 Apr 30;25(9):4907. doi: 10.3390/ijms25094907 (PMC11084865; doi:10.3390/ijms25094907)
Supplement: Supplementary file 1 [file ijms-25-04907-s001.zip › ijms-2913855-supplementary/Supplementary files/Supplementary Figure 1 and 2 (revised Uezono).pptx]

## Slide 1
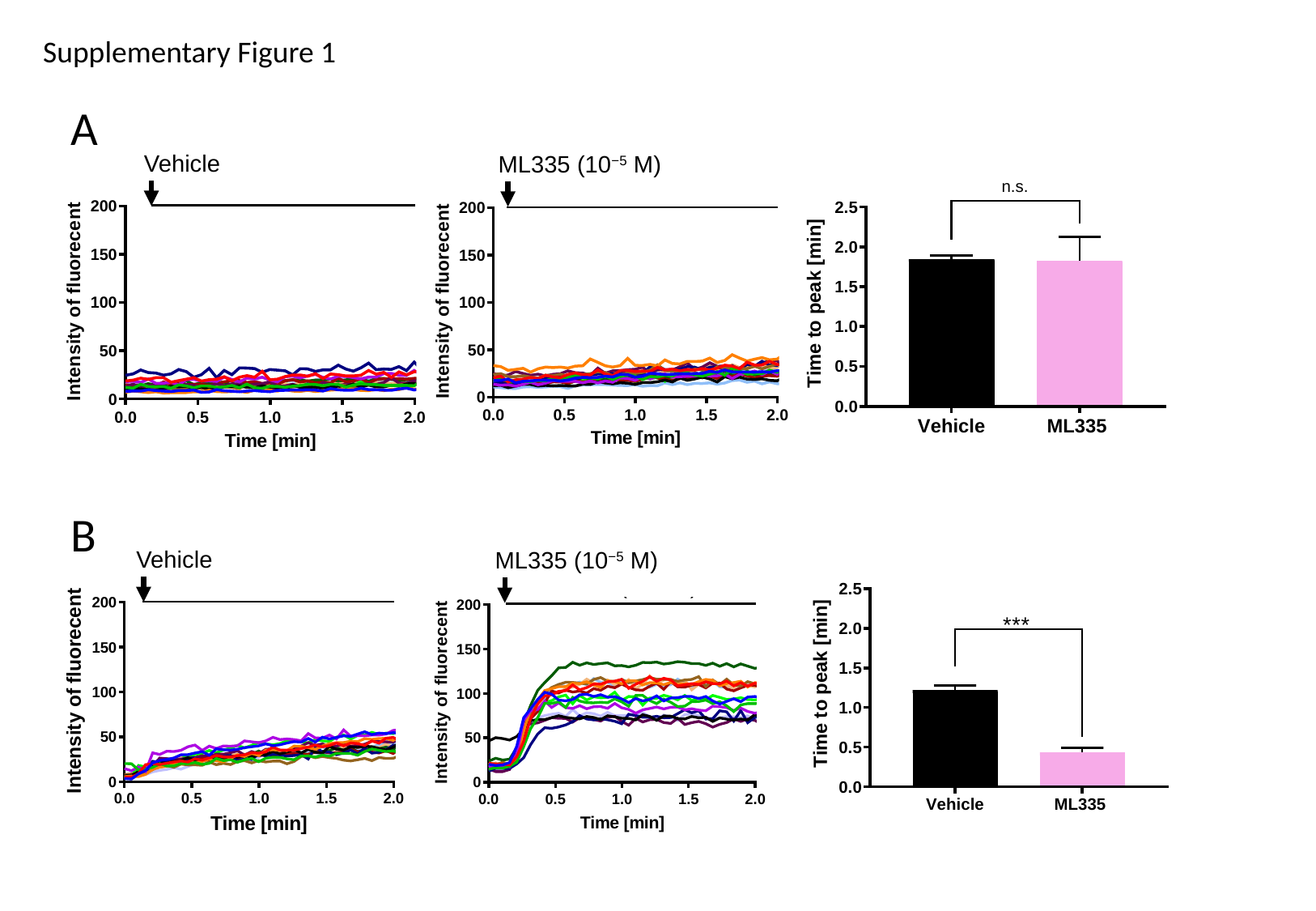

Supplementary Figure 1
A
Vehicle
ML335 (10−5 M)
B
Vehicle
ML335 (10−5 M)

## Slide 2
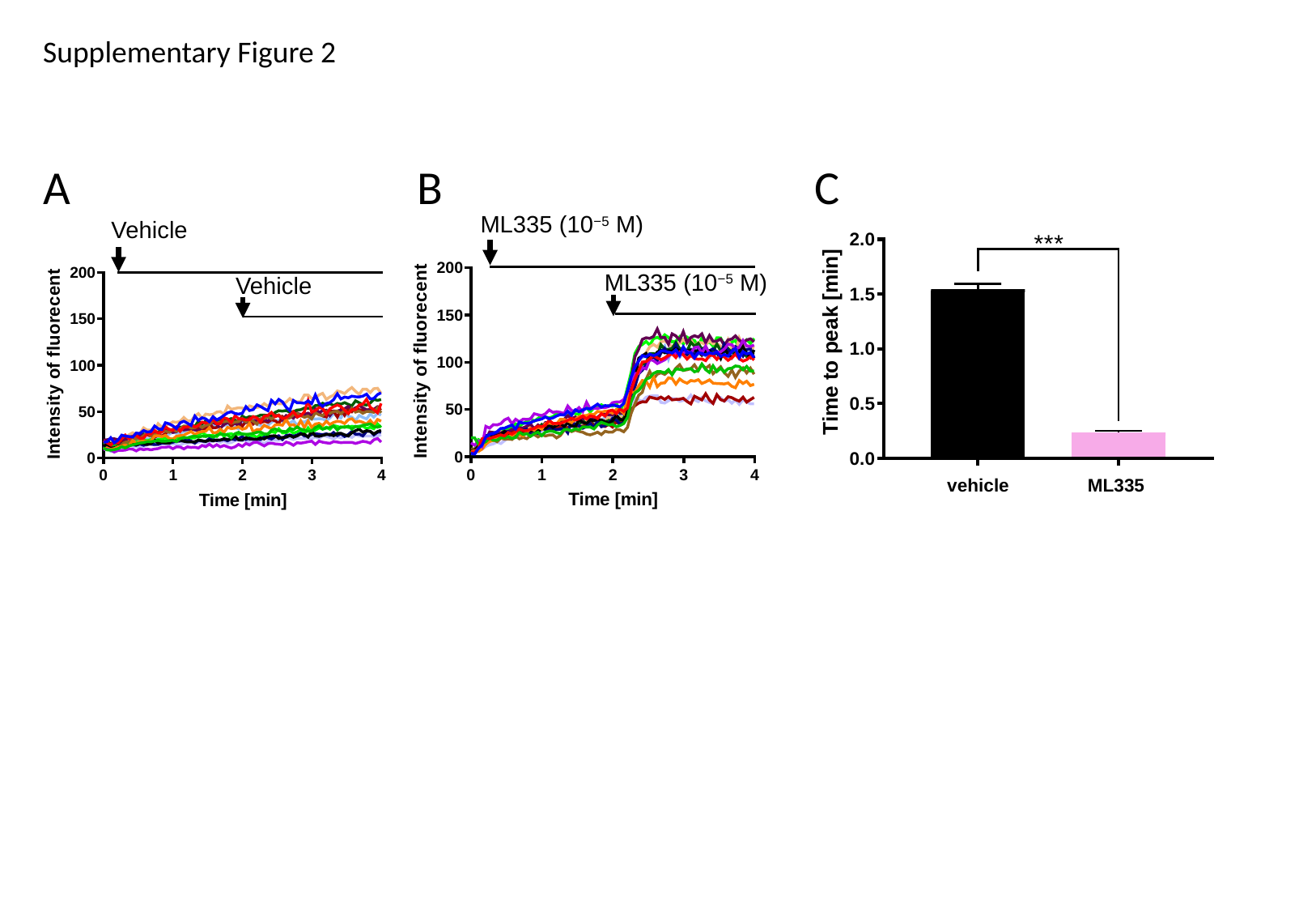

Supplementary Figure 2
A
B
C
ML335 (10−5 M)
Vehicle
ML335 (10−5 M)
Vehicle
