# Supplementary material for: The Inhibition of TREK-1 K+ Channels via Multiple Compounds Contained in the Six Kamikihito Components, Potentially Stimulating Oxytocin Neuron Pathways
Source: Int J Mol Sci. 2024 Apr 30;25(9):4907. doi: 10.3390/ijms25094907 (PMC11084865; doi:10.3390/ijms25094907)
Supplement: Supplementary file 1 [file ijms-25-04907-s001.zip › ijms-2913855-supplementary/Supplementary files/Supplementary Figure legends (revised by Uezono).pdf]

Supplementary Figure legends

Supplementary Figure 1. Effects of a TREK-1 agonist ML335 in HEK293 cells or TREK-1-expressing HEK293 cells. (A) HEK293 cells were treated with vehicle or ML335 (10^{-5} M) and intensity of fluorescent were measured using a FluxOR™ potassium ion channel assay. Representative tracing of the fluorescence intensity in randomly selected HEK293 cells treated with vehicle (*n* = 15, the right figure) or ML-335 (*n* = 15, the center figure). The activity of TREK-1 was estimated using the time to peak fluorescence following vehicle (*n* = 63) or ML335 (*n* = 39) treatment (the left figure). (B) Representative tracing of the fluorescence intensity in randomly selected TREK-1-expressing HEK293 cells treated with vehicle (*n* = 15, the right figure) or ML-335 (*n* = 15, the center figure). The activity of TREK-1 was estimated using the time to peak fluorescence following vehicle (*n* = 102) or ML335 (*n* = 105) treatment (the left figure). The data were expressed as the mean ± SEM. *** indicated $p < 0.001$, compared with vehicle; Mann-Whitney test.

Supplementary Figure 2. Effects of a TREK-1 agonist ML335 in TREK-1-expressing HEK293 cells using a FluxOR™ potassium ion channel assay. The cells were pretreated with vehicle for 2 min and subsequently treated with vehicle or ML335 (10^{-5} M). Representative tracing of the fluorescence intensity in randomly selected HEK293 cells treated with vehicle (*n* = 15, A) or ML-335 (*n* = 15, B). The activity of TREK-1 was estimated using the time to peak fluorescence following 2nd vehicle (*n* = 98) or 2nd ML335 (*n* = 119) treatment (C). The data were expressed as the mean ± SEM. *** indicated $p < 0.001$, compared with vehicle; Mann-Whitney test.
